# Supplementary material for: Bow shock oscillations of Mars under weakly disturbed solar wind conditions
Source: Nat Commun. 2025 Oct 31;16:9649. doi: 10.1038/s41467-025-65011-8 (PMC12578890; doi:10.1038/s41467-025-65011-8)
Supplement: Supplementary file 1 — Supplementary Information [file 41467_2025_65011_MOESM1_ESM.pdf]

Supplementary Information for

Bow Shock Oscillations of Mars Under Weakly Disturbed Solar Wind Conditions

Long Cheng[1,2,3], Yuming Wang[1,3,4,*], Yingjuan Ma[5], Robert Lillis[2], Jasper Halekas[6], Benoit Langlais[7], Tielong Zhang[3,8,9], Aibing Zhang[10,11], Guoqiang Wang[9], Sudong Xiao[9], Zhuxuan Zou[1,3], Yutian Chi[12], Xinjun Hao[1,3], Yiren Li[1,3], Zonghao Pan[1,3], Kai Liu[1,3]

[1] National Key Laboratory of Deep Space Exploration/School of Earth and Space Sciences, University of Science and Technology of China, Hefei, China
[2] Space Sciences Laboratory, University of California, Berkeley, Berkeley, CA, USA
[3] CAS Center for Excellence in Comparative Planetology/CAS Key Laboratory of Geospace Environment/Mengcheng National Geophysical Observatory, University of Science and Technology of China, Hefei, China
[4] Hefei National Laboratory, University of Science and Technology of China, Hefei, China
[5] Department of Earth, Planetary, and Space Sciences, University of California Los Angeles, Los Angeles, CA, USA
[6] Department of Physics and Astronomy, University of Iowa, Iowa City, Iowa, USA
[7] Nantes Université, Univ Angers, Le Mans Université, CNRS, Laboratoire de Planétologie et Géosciences, LPG UMR 6112, 44000 Nantes, France
[8] Space Research Institute, Austrian Academy of Sciences, Graz, Austria
[9] Institute of Space Science and Applied Technology, Harbin Institute of Technology, Shenzhen, China
[10] National Space Science Center, Chinese Academy of Sciences, Beijing, China
[11] University of Chinese Academy of Sciences, Beijing, China
[12] Institute of Deep Space Sciences, Deep Space Exploration Laboratory, Hefei, China
[*] Corresponding author. Email: ymwang@ustc.edu.cn

This file includes:

- Supplementary Notes
- Supplementary Figures 1−7
- Supplementary Tables 1−4
- Supplementary References

Supplementary Notes

1. BS crossings

Tianwen-1 was launched on 23 July 2020, and went into Martian orbit on 10 February 2021. It entered the final scientific orbit on 8 November 2021, starting the global scientific exploration of the planet. Tianwen-1 is a large elliptical orbit with a periareon of ~265 km and an apoareon altitude of ~12000 km, which allows the in situ measurements in various regions of the magnetosphere. MOMAG is the magnetometer onboard Tianwen-1 orbiter[1,2], which began to collect data on 13 November 2021. The instrument, combined with the ion analyzer onboard Tianwen-1 and the magnetometer and plasma instruments onboard MAVEN, provide two-point observations of the magnetic field and plasma environment of the upstream solar wind and Martian magnetosphere.

In January 2022, Tianwen-1's orbit was gradually sinked into the sheath region (see Supplementary Fig. 1). Thus, we focus on the period from 13 November to 31 December in 2021 when both Tianwen-1 and MAVEN's orbits repeatedly crossed between the solar wind and the Martian magnetosphere and recorded the BS crossing in the magnetic field and ion data. Typically, the spacecraft crossed the BS one time during an inbound or outbound one-way trajectory, as shown by the example in Supplementary Fig. 2a. The BS can be identified by the notable differences of magnetic field and ion spectrum between the up and down-streams. The IMF strength in the upstream of the BS was typically 2-5 nT, while the magnetic field strength in the downstream was higher and more fluctuating, as the magnetic field drapped in the magnetosheath. The solar wind proton is also shocked and heated in the magnetosheath.

Tianwen-1 also recorded some multi-crossing BS events, which may reveal the oscillation of the BS. Supplementary Fig. 2b shows a triple-crossing case, as marked by the dashed vertical lines. The strength and components of the magnetic field sharply changed between the levels of the solar wind and the sheath. During the period of this case, the solar wind ion flux was weak in MINPA, due to the deflection of the solar wind beam toward $-Y$ direction in Mars-Solar-Orbital (MSO) coordinate system (see the last subsection of Methods), but the proton energy spectra still clearly showed the transition between the solar wind and magnetosheath.

2. Transients near the BS

Transient phenomena near the BS[3,4] mainly include: 1) hot flow anomalies, spontaneous hot flow anomalies, foreshock bubbles, foreshock cavities, foreshock cavitons, density holes, foreshock compressional boundaries, short large-amplitude magnetic structures (SLAMS) in the foreshock, 2) high speed jets[5,6] and magnetic holes in the magnetosheath.

Hot Flow Anomalies (HFAs) are local transient structure at the BS that contain low field strength and low density core with depleted and heated plasma, which are typically driven by solar wind tangential discontinuities[4,7]. Spontaneous Hot Flow Anomalies (SHFAs) are similar to HFAs but formed in the absence of any solar wind discontinuities. Foreshock bubbles (FBs) are ion kinetic scale transient structures formed due to the interaction of backstreaming foreshock ions with a solar wind rotational discontinuity. FBs are characterized by a heated and tenuous core with significant flow deflection and a shock upstream of the core[4,8]. Foreshock cavities manifest distinctively as regions featuring low density and field strength core, bordered by high density and field strength compressional boundaries, but the flow deflection and

plasma heating within the cavities is weak. Foreshock cavitons are similar to foreshock cavities, but without clear heating and flow deflection. Foreshock compressional boundaries (FCBs) emerge along the interface between the foreshock and the solar wind, associated with enhanced density and field strength, occasionally accompanied with density and field strength depletion on the foreshock side[4]. Short Large-Amplitude Magnetic Structures (SLAMS) are characterized by their short duration and significant amplitude variations in the magnetic field[4]. Density holes are characterized by similarly shaped magnetic holes with enhanced density and field strength at one or both edges[4].

Magnetosheath jets, also termed high speed jets, are high speed structures with significant enhanced dynamic pressure in the magnetosheath, which can be due to an increased density and/or velocity[5]. Magnetic holes have an observable magnetic field decrease in a short time span, which are associated with increased density or temperature, as pressure balance structures[9].

Schematic illustrations of those transient phenomena are shown in Fig. 2. Characteristics of different transients in the magnetic field and ion energy spectra are shown in Table 1, which could be measured by MOMAG and MINPA of Tianwen-1.

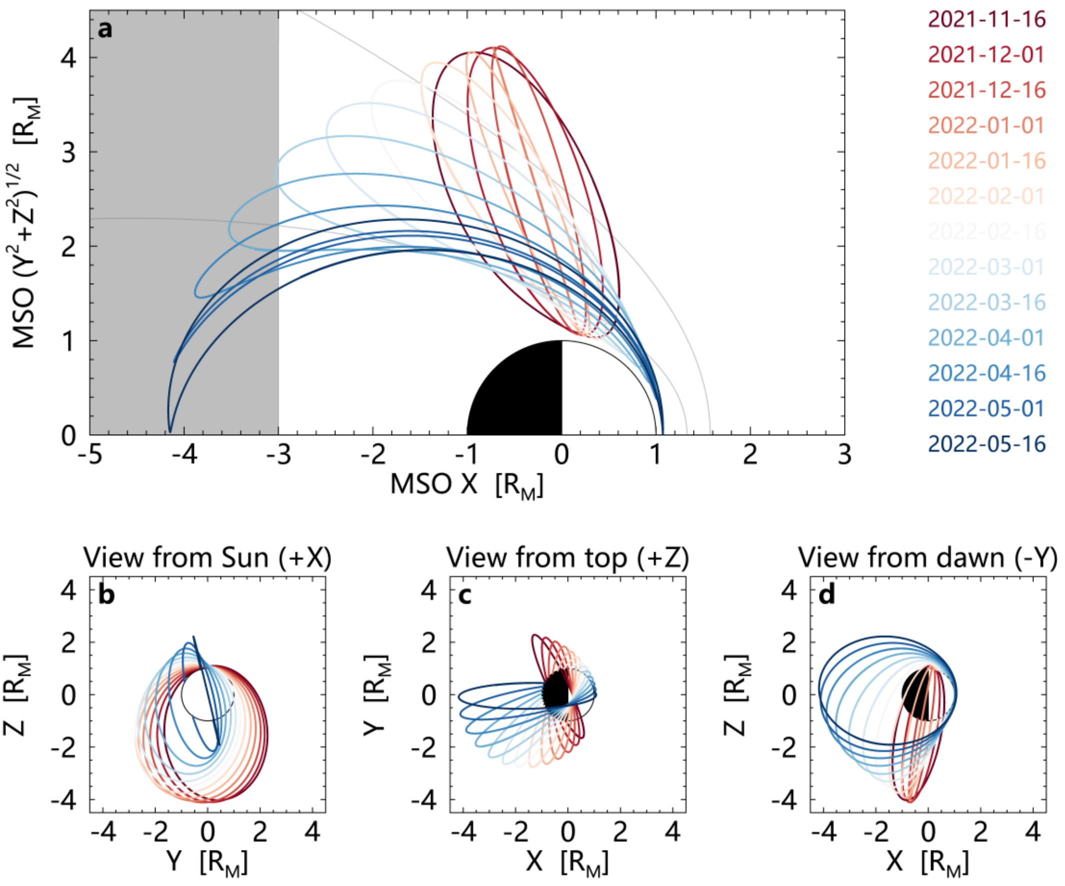

Supplementary Fig. 1 | Orbits of Tianwen-1 from November 2021 to May 2022.

a Tianwen-1 orbits from November 2021 to May 2022 in the cylindrical Mars-Solar-Orbital coordinates, where locations of the bow shock and magnetic pileup boundary by Edberg et al.[10] are shown for reference and the gray box represents the region with X<-3 R_M. **b** Tianwen-1 orbits in Y-Z plane, as viewed from Sun (+X). **c** Tianwen-1 orbits in X-Y plane, as viewed from the top (+Z). **d** Tianwen-1 orbits in X-Z plane, as viewed from the dawn (-Y).

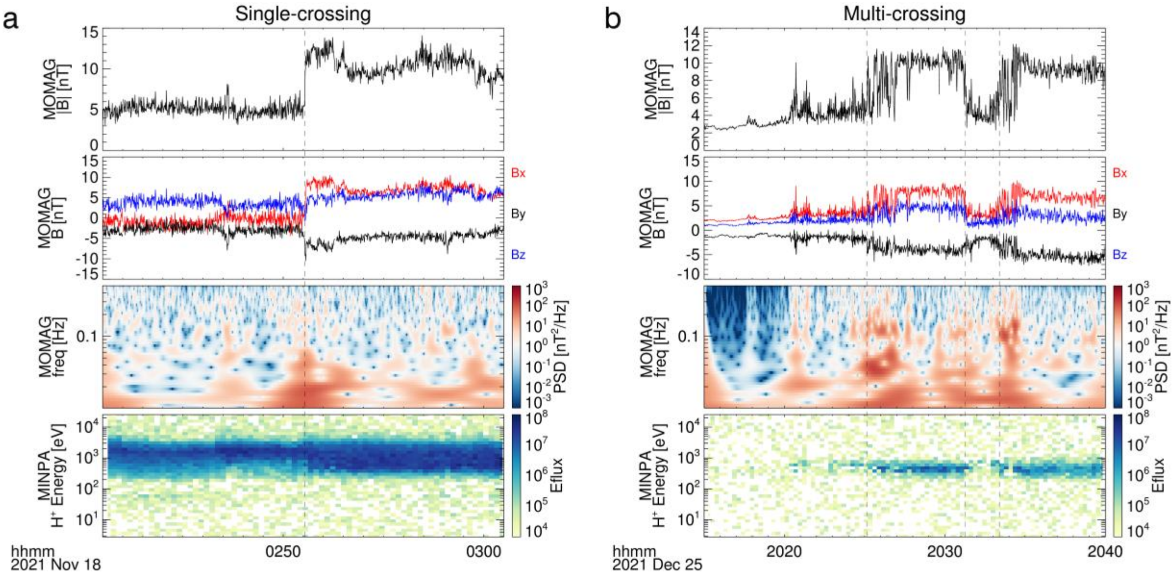

Supplementary Fig. 2 | Examples of single-crossing and multi-crossing BS events of Tianwen-1.

a a single-crossing BS event of Tianwen-1, where the panels show the variations of |**B**|, the components of the magnetic field in Mars-Solar-Orbital coordinates, the wavelet transform of |**B**| with colors represent the power spectral density (PSD) in nT^2 Hz^{-1}, and the proton energy spectra with colors represent the energy flux in eV/(eV·cm^2·s·sr). **b** similar to **a** but for a multi-crossing BS event. The vertical dashed lines denote the BS crossings.

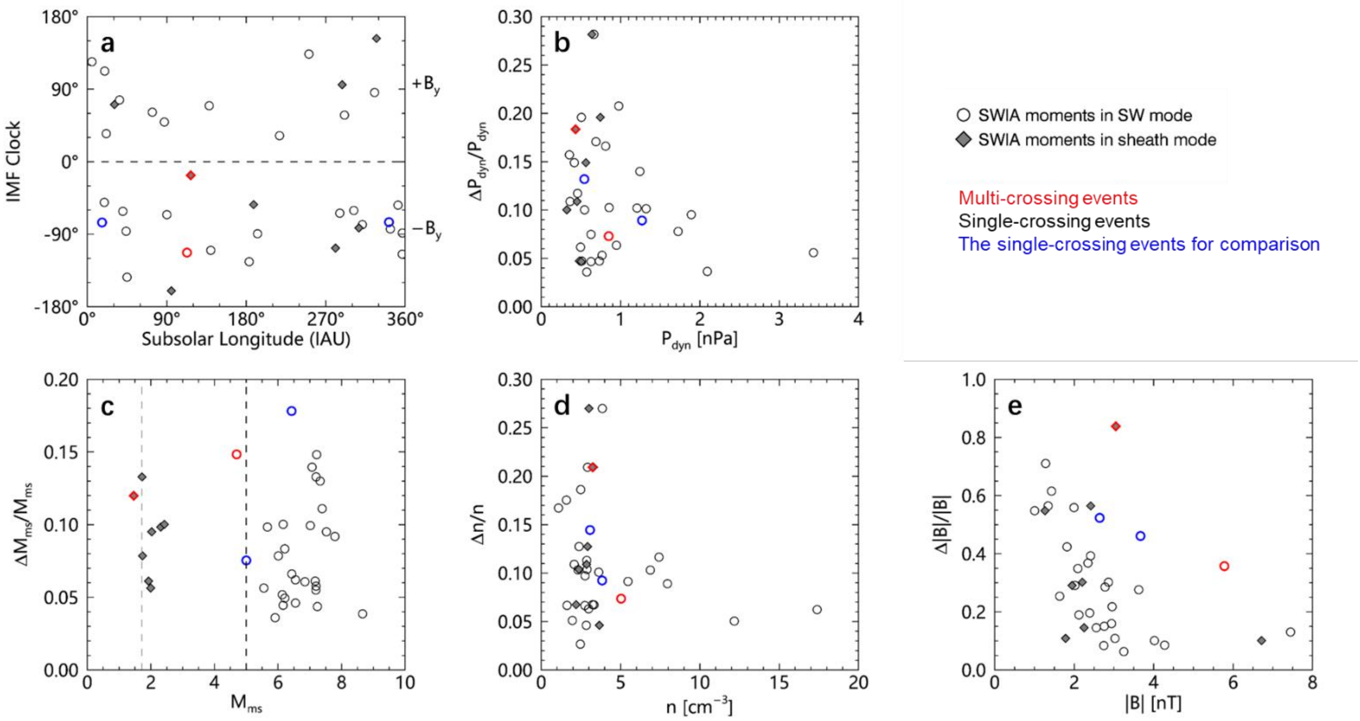

Supplementary Fig. 3 | Upstream solar wind and IMF parameters.

a scatter plot of the IMF clock angles versus subsolar longitudes. **b-e** the disturbance levels of the solar wind dynamic pressure, magnetosonic Mach number, density, and IMF strength during the events of interest. Source data are provided as a Source Data file.

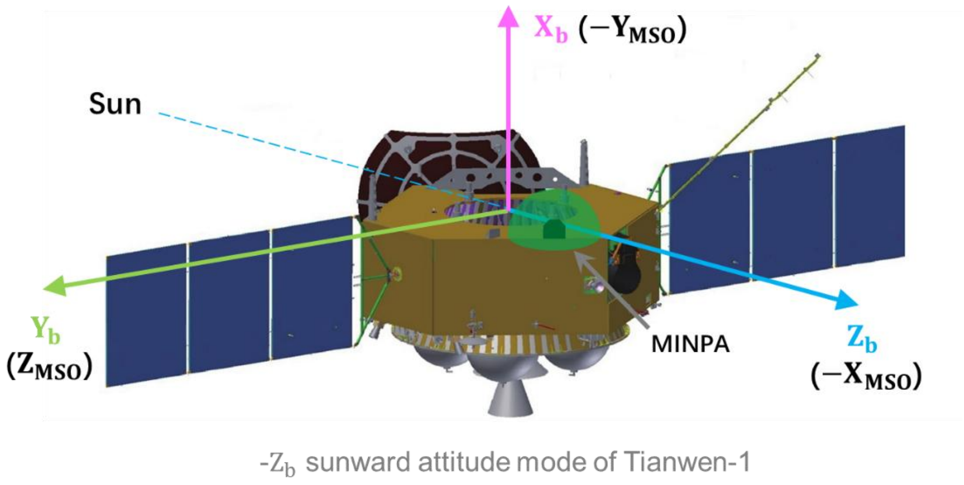

Supplementary Fig. 4 | Schematic illustrations of the geometry of Tianwen-1/MINPA. Location of MINPA onboard Tianwen-1 orbiter and its attitude and field of view (FOV) in the solar wind, where X_b, Y_b and Z_b represent the axes of the orbiter coordinate system and X_{MSO}, Y_{MSO} and Z_{MSO} represent the axes of the Mars-Solar-Orbital (MSO) coordinate system. The green half sphere illustrates the 360°×90° FOV of MINPA ion measurement. The figure is adapted from Figure 1 in the work of Chen et al.[11].

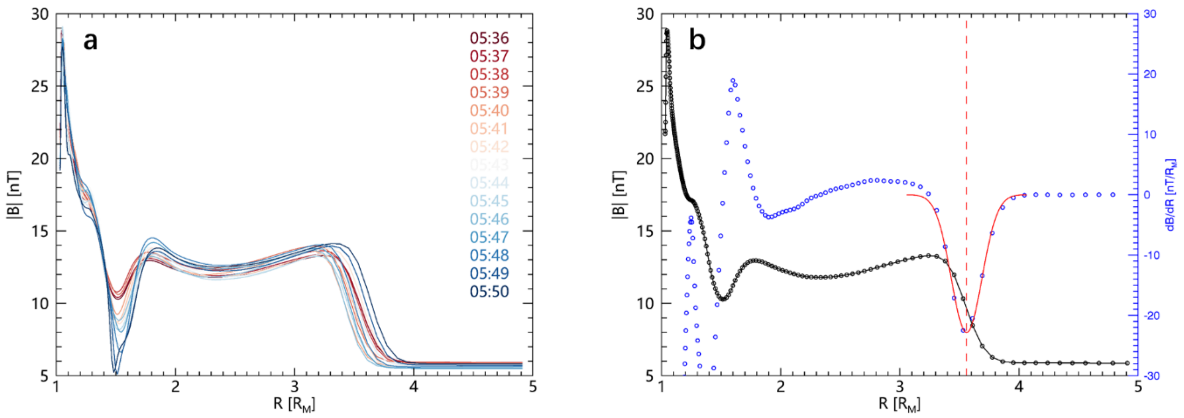

Supplementary Fig. 5 | Example of automated identification of the simulated bow shock location.

a magnetic field profiles along the radial direction passing through the vicinity of Tianwen-1. **b** the radial profile of the magnetic field at a given time and the corresponding gradient of the magnetic field, with the red solid line representing a Gaussian fit to data points near the BS location and vertical dashed line indicating the identified BS location.

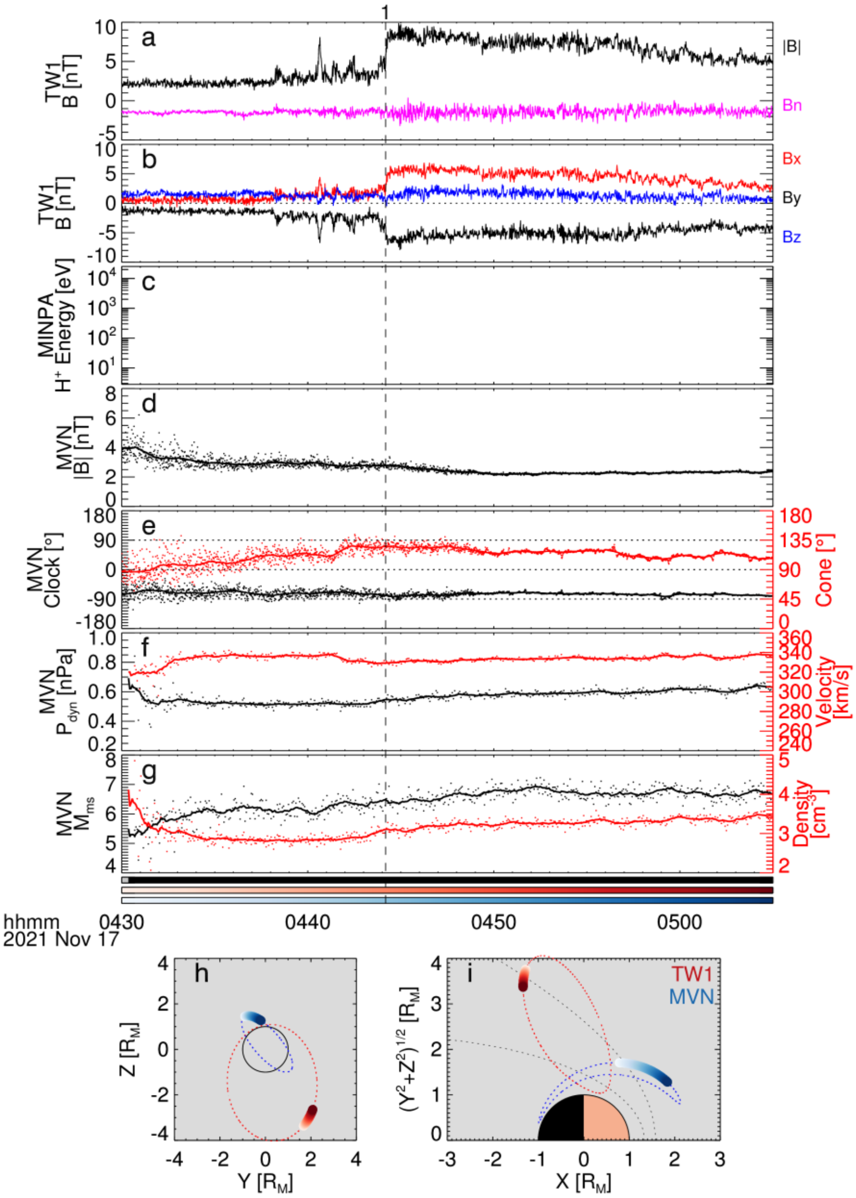

Supplementary Fig. 6 | Observations of the single-crossing Event 12 on 2021 November 17. **a** the magnetic field strength (black) from MOMAG 1Hz data and the normal magnetic field (magenta). **b** three components of the magnetic field from MOMAG in MSO coordinates. **c** proton energy spectra measured by MINPA, with colors represent the energy flux in eV/(eV·cm^2·s·sr). **d** the magnetic field strength from MAVEN/MAG 1Hz data. **e** directions of the magnetic field from MAVEN/MAG. **f** the solar wind dynamic pressure and velocity. **g** the magnetosonic Mach number and density. **h** positions of Tianwen-1 and MAVEN in the MSO *Z*-*Y* plane. **i** positions of Tianwen-1 and MAVEN in the MSO cylindrical coordinates. The layout and symbols are the same as those in Figure 1.

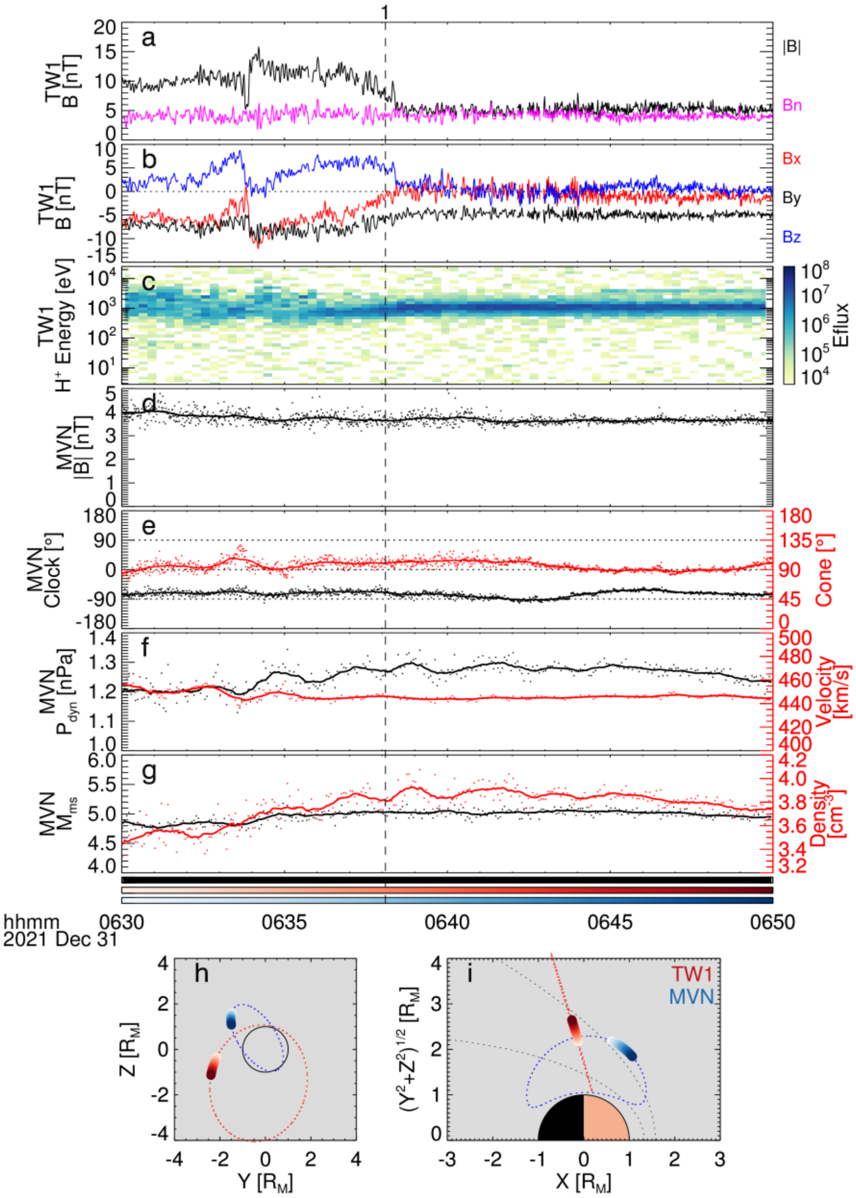

Supplementary Fig. 7 | Observations of the single-crossing Event 37 on 2021 December 31. **a** the magnetic field strength (black) from MOMAG 1Hz data and the normal magnetic field (magenta). **b** three components of the magnetic field from MOMAG in MSO coordinates. **c** proton energy spectra measured by MINPA, with colors represent the energy flux in eV/(eV·cm^2·s·sr). **d** the magnetic field strength from MAVEN/MAG 1Hz data. **e** directions of the magnetic field from MAVEN/MAG. **f** the solar wind dynamic pressure and velocity. **g** the magnetosonic Mach number and density. **h** positions of Tianwen-1 and MAVEN in the MSO *Z*-*Y* plane. **i** positions of Tianwen-1 and MAVEN in the MSO cylindrical coordinates. The layout and symbols are the same as those in Figure 1.

Supplementary Table 1 | Shock normal in Event 1.

The normal are calculated by the minimum variance analysis, using the magnetic field data within the listed time window. $\hat{\mathbf{n}}$ represents the normal in the entire event, while $\hat{\mathbf{n}}_1$, $\hat{\mathbf{n}}_2$ and $\hat{\mathbf{n}}_3$ represent the normal of each crossing.

	Time Window	Value	Intersection Angle			
			$\hat{\mathbf{n}}_1$	$\hat{\mathbf{n}}_2$	$\hat{\mathbf{n}}_3$	$\hat{\mathbf{n}}$
$\hat{\mathbf{n}}_1$	05:34:00 ~ 05:38:00	[0.56, 0.17, -0.81]		6.3°	5.0°	2.2°
$\hat{\mathbf{n}}_2$	05:38:00 ~ 05:41:00	[0.60, 0.07, -0.79]	6.3°		1.4°	8.4°
$\hat{\mathbf{n}}_3$	05:41:00 ~ 05:42:15	[0.60, 0.10, -0.79]	5.0°	1.4°		7.1°
$\hat{\mathbf{n}}$	05:25:00 ~ 05:50:00	[0.53, 0.20, -0.82]	2.2°	8.4°	7.1°	

Supplementary Table 2 | Shock normal in Event 2.

Similar to Supplementary Table 1, but for Event 2.

	Time Window	Value	Intersection Angle			
			$\hat{\mathbf{n}}_1$	$\hat{\mathbf{n}}_2$	$\hat{\mathbf{n}}_3$	$\hat{\mathbf{n}}$
$\hat{\mathbf{n}}_1$	20:23:00 ~ 20:28:00	[0.58, 0.34, -0.74]		4.1°	1.6°	1.2°
$\hat{\mathbf{n}}_2$	20:28:00 ~ 20:32:30	[0.58, 0.40, -0.71]	4.1°		4.1°	3.3°
$\hat{\mathbf{n}}_3$	20:32:30 ~ 20:36:00	[0.56, 0.35, -0.75]	1.6°	4.1°		2.4°
$\hat{\mathbf{n}}$	20:15:00 ~ 20:40:00	[0.59, 0.34, -0.73]	1.2°	3.3°	2.4°	

Supplementary Table 3 | Related parameters of the IMF and BS normal.

All vectors are in MSO coordinates. The value of IMF vector are measured by the MAVEN/MAG. Calculations of the BS normal from the MVA and the BS model by Edberg et al.[10] are described in Methods. Event 1 and Event 2 represents the BS events shown in Fig. 1 and Fig. 3 respectively.

Parameter	Event 1	Event 2
IMF (nT)	(3.31, -4.37, -1.27)	(2.40, -0.52, 1.50)
BS normal from the MVA, $\mathbf{n}_{\mathrm{MVA}}$	(0.53, 0.20, -0.82)	(0.59, 0.34, -0.73)
BS normal from the model, $\mathbf{n}_{\mathrm{mod}}$	(0.60, 0.25, -0.76)	(0.62, 0.26, -0.74)
Angle between $\mathbf{n}_{\mathrm{MVA}}$ and $\mathbf{n}_{\mathrm{mod}}$ (°)	5.7	5.4
Angle between IMF and $\mathbf{n}_{\mathrm{MVA}}$, θ_{Bn} (°)	69.9	87.1

Supplementary Table 4 | List of periods of bow shock events of interest and corresponding solar wind parameters, based on 60-second smoothed data measured by MAVEN in the solar wind.

No.	Start Time (UT)	End Time (UT)	N (cm^{-3})	P_{dyn} (nPa)	M_{ms}
Multi-crossing events					
1	2021-12-02/05:25:00	2021-12-02/05:50:00	5.03	0.85	4.70
2*	2021-12-25/20:15:00	2021-12-25/20:40:00	3.24	0.43	1.46
Single-crossing events					
3*	2021-11-13/18:50:00	2021-11-13/19:10:00	2.37	0.32	1.93
4	2021-11-14/01:30:00	2021-11-14/02:00:00	2.88	0.55	7.17
5	2021-11-14/23:00:00	2021-11-14/23:20:00	3.85	0.66	7.20
6	2021-11-15/03:10:00	2021-11-15/03:30:00	2.38	0.42	6.01
7	2021-11-15/06:00:00	2021-11-15/06:20:00	2.92	0.51	7.52
8	2021-11-15/20:30:00	2021-11-15/20:50:00	2.08	0.36	5.67
9	2021-11-16/00:00:00	2021-11-16/00:10:00	2.84	0.51	6.16
10	2021-11-16/14:10:00	2021-11-16/14:25:00	3.24	0.63	5.55
11	2021-11-16/17:50:00	2021-11-16/18:00:00	3.33	0.63	6.13
12	2021-11-17/04:40:00	2021-11-17/04:50:00	3.07	0.54	6.43
13	2021-11-18/02:40:00	2021-11-18/03:00:00	7.95	1.73	6.55
14	2021-11-20/20:30:00	2021-11-20/20:50:00	2.49	0.98	7.22
15	2021-11-21/03:30:00	2021-11-21/03:45:00	1.96	0.95	5.91
16	2021-11-21/10:40:00	2021-11-21/11:00:00	1.59	0.69	6.43
17*	2021-11-23/05:10:00	2021-11-23/05:30:00	3.01	0.64	1.72
18*	2021-11-23/09:30:00	2021-11-23/09:50:00	2.92	0.56	1.74
19*	2021-11-23/12:10:00	2021-11-23/12:30:00	3.30	0.74	2.03
20	2021-11-23/19:25:00	2021-11-23/19:40:00	2.99	0.77	7.24
21	2021-11-25/18:20:00	2021-11/25/18:40:00	5.47	1.21	7.39
22	2021-11-29/09:45:00	2021-11-29/10:05:00	2.31	0.46	7.32
23*	2021-11-29/16:35:00	2021-11-29/17:00:00	2.86	0.45	2.31
24	2021-11-30/14:15:00	2021-11-30/14:35:00	2.75	0.50	6.16
25*	2021-12-01/16:05:00	2021-12-01/16:30:00	3.66	0.48	2.43
26	2021-12-02/13:45:00	2021-12-02/14:05:00	17.39	3.43	6.21
27	2021-12-07/16:05:00	2021-12-07/16:30:00	1.09	0.35	7.07
28	2021-12-08/20:30:00	2021-12-08/20:45:00	6.88	1.89	7.19
29*	2021-12-11/06:15:00	2021-12-11/06:30:00	2.19	0.50	1.99
30	2021-12-14/18:00:00	2021-12-14/18:20:00	2.47	0.57	7.20
31	2021-12-15/22:00:00	2021-12-15/22:15:00	7.42	1.24	7.79
32	2021-12-16/05:15:00	2021-12-16/05:30:00	12.17	2.09	8.66
33	2021-12-17/21:45:00	2021-12-17/22:00:00	2.77	1.32	6.55
34	2021-12-18/18:40:00	2021-12-18/19:00:00	1.63	0.73	6.85
35	2021-12-21/19:45:00	2021-12-21/20:00:00	2.87	0.81	7.02
36	2021-12-23/07:30:00	2021-12-23/07:40:00	3.62	0.86	6.21
37	2021-12-31/06:30:00	2021-12-31/06:45:00	3.84	1.27	5.00

Note: * denotes SWIA moments in sheath mode; all others are in solar wind mode.

Supplementary References

1. Wang, Y. *et al.* The Mars orbiter magnetometer of Tianwen-1: in-flight performance and first science results. *E&PP* **7**, 1–13 (2023).
2. Zou, Z. *et al.* In-flight Calibration of the Magnetometer on the Mars Orbiter of Tianwen-1. *Sci. China Technol. Sci.* **66**, 2396–2405 (2023).
3. Zhang, H. & Zong, Q. Transient phenomena at the magnetopause and bow shock and their ground signatures: Summary of the geospace environment modeling (GEM) focus group findings between 2012 and 2016. in *Dayside Magnetosphere Interactions* 13–37 (John Wiley & Sons, Inc., 2020).
4. Zhang, H. *et al.* Dayside Transient Phenomena and Their Impact on the Magnetosphere and Ionosphere. *SSRv* **218**, 40 (2022).
5. Plaschke, F. *et al.* Jets Downstream of Collisionless Shocks. *SSRv* **214**, 81 (2018).
6. Gunell, H., Hamrin, M., Nesbit-Östman, S., Krämer, E. & Nilsson, H. Magnetosheath jets at Mars. *SciA* **9**, eadg5703 (2023).
7. Collinson, G. *et al.* A hot flow anomaly at Mars. *GeoRL* **42**, 9121–9127 (2015).
8. Madanian, H. *et al.* Transient Foreshock Structures Upstream of Mars: Implications of the Small Martian Bow Shock. *GeoRL* **50**, e2022GL101734 (2023).
9. Madanian, H. *et al.* Magnetic Holes Upstream of the Martian Bow Shock: MAVEN Observations. *JGRA* **125**, e2019JA027198 (2020).
10. Edberg, N. J. T., Lester, M., Cowley, S. W. H. & Eriksson, A. I. Statistical analysis of the location of the Martian magnetic pileup boundary and bow shock and the influence of crustal magnetic fields. *JGRA* **113**, A08206 (2008).
11. Chen, M. *et al.* Deployable boom for Mars Orbiter Magnetometer onboard Tianwen-1. *J. Univ. Sci. Technol. China* **52**, 7 (2022).
