# Supplementary material for: Bow shock oscillations of Mars under weakly disturbed solar wind conditions
Source: Nat Commun. 2025 Oct 31;16:9649. doi: 10.1038/s41467-025-65011-8 (PMC12578890; doi:10.1038/s41467-025-65011-8)
Supplement: Supplementary file 2 — Description of Additional Supplementary Files [file 41467_2025_65011_MOESM2_ESM.pdf]

Description of Additional Supplementary Files

Supplementary Movie 1:

Description: The animation of the three-dimensional MHD numerical simulation of Event 1 showing the global oscillation of the Martian bow shock. The X-Y plane presents the solar wind velocity with the lower color bar and the X-Z plane the magnetic field strength with the upper color bar. The rough position of Tianwen-1 and MAVEN during the event are indicated by spacecraft images. The black region is Mars.
